# CRISPR/Cas9 screening identifies SUV39H2 as a key regulator of oHSV-1 resistance in oral squamous cell carcinoma

**DOI:** 10.1038/s41420-025-02702-7

**Published:** 2025-08-23

**Authors:** Manman Qiu, Qicheng Zhang, Rui Li, Rongrong Wei, Jiawei Zhao, Juan Tan, Hongkai Zhang, Wentao Qiao

**Affiliations:** 1 Key Laboratory of Molecular Microbiology and Technology, Ministry of Education, College of Life Sciences, Nankai University, Tianjin, China; 2 Tianjin Key Laboratory of Lung Cancer Metastasis and Tumor Microenvironment, Tianjin Lung Cancer Institute, Tianjin Medical University General Hospital, Tianjin, China; 3 School of Medicine, Nankai University, Tianjin, China; 4 Tianjin Key Laboratory of Protein Sciences, Department of Biochemistry and Molecular Biology, College of Life Sciences, Nankai University, Tianjin, China

**Keywords:** Oral cancer, Target validation

## Abstract

Oncolytic viruses represent an innovative strategy for cancer therapy. However, extensive gene expression reprogramming within tumor cells may hinder viral propagation by affecting essential cell-virus interactions. Here, through genome-wide CRISPR/Cas9 library screening, Suppressor of variegation 3–9 homolog 2 (SUV39H2), a histone methyltransferase, was identified as a critical factor in mediating resistance to oncolytic herpes simplex virus 1 (oHSV-1) in oral squamous cell carcinoma (OSCC). Functional studies in SCC15 cells revealed that SUV39H2 knockdown facilitated viral replication, while its overexpression suppressed it. The inhibitor OTS186935 targeting SUV39H2 was administered to evaluate its effects on viral replication both in vitro and in vivo. Pretreatment with OTS186935 in SCC15, SCC7, and MCF7 led to a significant enhancement of viral replication. Combined treatment with OTS186935 and oHSV-1 demonstrated significant anti-tumor efficacy in BALB/c nude mice bearing SCC15 tumors. SUV39H2 was shown to regulate the trimethylation of lysine 9 on histone 3 (H3K9me3) at the viral promoter regions of immediate-early gene *ICP0*, *ICP4* and early gene *ICP8*, thereby repressed viral gene transcription. However, oHSV-1 infection induced the degradation of SUV39H2, a process mediated by the viral protein ICP0 through the proteasomal pathway. Findings from studies in SCC7 cells further supported the observation that SUV39H2 knockdown enhanced viral replication. Moreover, SUV39H2 downregulation increased CD4+ and CD8+ T cell infiltration in syngeneic tumors treated with oHSV-1. TCGA database analysis revealed that SUV39H2 is associated with distinct immune cell infiltration patterns across different cancer types and correlates with immune checkpoint expression. These results highlight the role of SUV39H2 in regulating oHSV-1 replication and indicate that SUV39H2 may represent a potential target to improve the efficacy of oncolytic virotherapy.

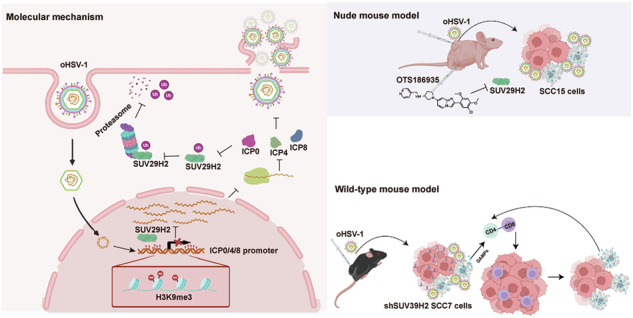

## Introduction

Oncolytic virotherapy has gained significant attention in clinical trials as a promising alternative to conventional cancer therapies, including treatment for OSCC [1]. OVs selectively infect and lyse tumor cells and stimulate systemic antitumor immune responses, enhancing their therapeutic efficacy [2–4]. Talimogene laherparepvec (T-VEC), the first FDA-approved oncolytic virotherapy for melanoma in 2015, has been genetically engineered to produce granulocyte-macrophage colony-stimulating factor (GM-CSF). This modification not only facilitates direct tumor lysis but also amplifies the host immune response against cancer cells [5, 6]. However, the emergence of resistance to OVs poses a significant challenge to the efficacy and sustainability of this treatment modality. Resistance mechanisms can manifest through several pathways, including the potential evolution of tumor cells to counteract the OVs by enhancing antiviral defenses or modifying cellular signaling pathways [7]. Furthermore, the tumor immune microenvironment (TME) may mount an immune response that neutralizes OVs before they can effectively target cancer cells [8–10]. Insufficient production of viral progeny reduces tumor cell death and weakens the activation of anti-tumor immune responses, highlighting the critical role of establishing infection and achieving robust replication in tumor cells for effective tumor destruction. Understanding the molecular and cellular underpinnings of OVs resistance is essential for developing strategies to overcome these barriers.

Oral squamous cell carcinoma (OSCC), a heterogeneous neoplasm arising from the mucosal lining of the oral cavity, is a prevalent form of head and neck malignancy, accounting for approximately 40% of all head and neck squamous cell carcinomas (HNSCC) [11–13]. Most patients are diagnosed at an advanced stage due to delayed detection. The conventional treatment for HNSCC involves a multimodal approach that combines surgery, radiation, and chemotherapy [14]. Despite advances in these therapies, 5-year survival rates remain at approximately 40-50% [15]. Furthermore, immunotherapy has emerged as a key strategy for the management of advanced tumors, particularly following the U.S. Food and Drug Administration’s (FDA) approval of immune checkpoint inhibitors (ICIs) in 2011 [16, 17]. However, treatment with ICIs alone does not always achieve curative outcomes. Consequently, new treatment approaches, including oncolytic viruses (OVs), vaccine therapies, and chimeric antigen receptor (CAR) T cells, are under active investigation [18–20]. Among them, OV therapy has gained significant attention, but certain cases of HNSCC have been found to exhibit resistance to this treatment [21].

Post-translational modifications (PTMs) on histones not only regulate transcription but also impact various other DNA processes, including repair, replication, and recombination [22, 23]. Histone methylation, occurring primarily on lysine and arginine residues of histones H3 and H4, is one of the most well-characterized and specific forms of histone modification ^22^. Nucleosome positions on the HSV-1 genome have been mapped via high throughput sequencing [24], and the histone modifications are detected on the HSV-1 genome in THP-1 cells, including H3K9me3, H3K27me3, H3K4me3 and H3K27ac [25]. H3K9me3 is considered the epigenetic hallmark of heterochromatin, a repressive chromatin state that may restrict viral gene expression and replication. Suppressor of variegation 3–9 homolog 2 (SUV39H2), the second H3K9-selective histone methyltransferase (HMT), specifically catalyzes H3K9me3 leading to gene silencing [26–28]. Accruing evidence suggests that SUV39H2 plays a significant role in cancer initiation and progression [29]. To date, rarer research has directly explored the role of SUV39H2 in viral replication.

CRISPR screening has proven to be a powerful tool for identifying genes that are critical to cancer cell resistance against drug treatments in various models [30–32] or for identifying host factors associated with infection by various viruses [33–35]. In this study, SUV39H2 was identified as a key regulator of oHSV-1 resistance in OSCC. SUV39H2 suppresses oHSV-1 replication by enriching H3K9me3 methylation at the promoter regions of key viral genes, thereby limiting viral transcription and replication. Notably, the viral protein ICP0 counteracts this suppression by targeting SUV39H2 for degradation via the proteasomal pathway. Furthermore, SUV39H2 inhibition significantly enhances oHSV-1 replication within tumor tissues and promotes CD4+ T and CD8+ T immune cell infiltration into the tumor microenvironment, ultimately improving the virus’s oncolytic efficacy. These findings suggest that targeting SUV39H2 represents a promising strategy to overcome oHSV-1 resistance in OSCC, providing potential for combination therapies in anti-tumor treatment and for identifying biomarkers that could predict the efficiency of oHSV-1 replication in tumors.

## Result

### CRISPR/Cas9 screening identifies SUV39H2 as a key regulator of oHSV-1 resistance in OSCC

**Fig. 1:**
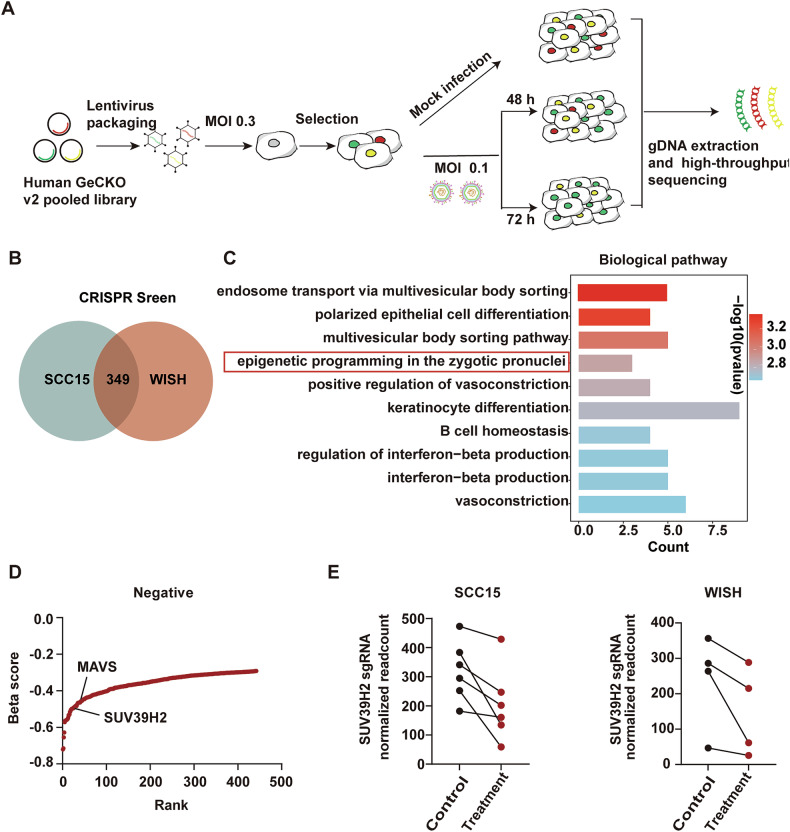
CRISPR library screening identified SUV39H2 as a driver for oHSV-1 resistance. **A** A schematic diagram illustrates the workflow of genome-wide CRISPR/Cas9 knockout library screening. The human genome-wide CRISPR/Cas9 knockout libraries A and B were packaged into lentiviral particles and transduced into SCC15 cells at an MOI of 0.3. The sgRNA-transduced cells were selected with puromycin to generate a mutant cell pool. Mutant cells were then cultured with oHSV-1 for 48 and 72 h for genetic screening. Genomic DNA was extracted from the treated cells, and the sgRNA fragments were amplified by PCR. The copy number of sgRNAs was determined by high-throughput sequencing and analyzed using the MAGeCK v0.5.7 algorithm. **B** A Venn diagram illustrates the overlap of genes identified in the genome-wide CRISPR/Cas9 knockout library screening between SCC15 cells infected with oHSV-1 and WISH cells infected with HSV-1. **C** GO analysis was performed on the overlap of genes. **D** The rankings of SUV39H2 and MAVS in the negative screening are indicated by the black line. **E** The sgRNAs targeting SUV39H2 were consistently depleted in Treatment group.

A genome-wide CRISPR/Cas9 knockout screen was conducted to identify key regulators of oHSV-1 resistance. The human GeCKO v2 CRISPR library (A and B) comprises 122,411 unique sgRNAs, targeting 19,052 protein-coding genes and 1864 microRNAs. The library was packaged into lentiviral particles to create a mutant cell pool in SCC15 cells. Cells were exposed to oHSV-1 for 48 (Control group) and 72 h (Treatmet group) to facilitate both positive and negative selection (Fig. 1A). An analysis of post-selection revealed approximately 500× coverage, with 99.52% (library A) and 99.88% (library B) sgRNA retention, ensuring sufficient depth and coverage. Additionally, CRISPR/Cas9 screening data (GSE223085) from HSV-1-infected human amnion WISH cells were retrieved from the GEO database. Genes exhibiting significant sgRNA depletion under selective pressure were identified as potential mediators of oHSV-1 resistance [36].

As shown in Fig. 1B, a total of 349 common genes were identified in the two independent negative-selection CRISPR/Cas9 screens. Gene Ontology (GO) enrichment analysis was conducted on the 349 intersecting genes. The analysis revealed notable enrichment in several key biological processes. Importantly, pathways related to immune response modulation, including interferon-beta production, B cell homeostasis, and regulation of interferon-beta production were also enriched, suggesting a role in antiviral defense mechanisms (Fig. 1C). Given the crucial role of histone modifications in tumor progression and the therapeutic potential of epigenetic inhibitors, we focused on histone-modifying enzymes [37]. Thus, we prioritized epigenetic regulators in oHSV-1 infection. In this context, we identified SUV39H2, a histone methyltransferase, as a key candidate for further investigation. SUV39H2 ranked prominently in negative-selection screening, highlighting its potential role in HSV-1 infection (Fig. 1D). Notably, MAVS, a well-known antiviral protein involved in the innate immune response against HSV, was also identified. All SUV39H2-targeting sgRNAs were significantly reduced in the Treatment group, implying that SUV39H2 depletion could enhance SCC15 cells sensitivity to oncolytic virus therapy (Fig. 1E). The above results indicate that SUV39H2 may play an important role in oHSV-1 resistance.

### SUV39H2 knockdown promotes oHSV-1 replication in SCC15 cell

**Fig. 2:**
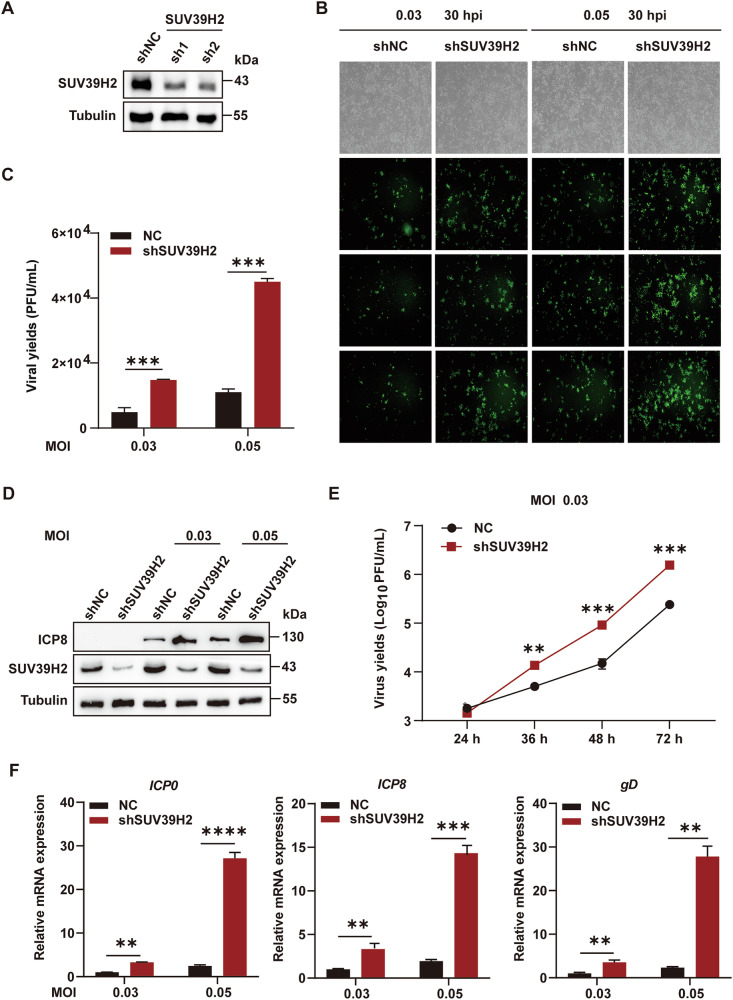
Effects of SUV39H2 knockdown on oHSV-1 replication in SCC15 cells. **A** Western blotting analysis demonstrated the efficiency of SUV39H2 knockdown in SCC15 cells following shRNA treatment. Tubulin was used as a loading control. **B** SCC15 cells were infected with oHSV-1 at MOIs of 0.03 and 0.05 and analyzed at 30 hpi. Fluorescence imaging of EGFP-positive cells showing the replication of oHSV-1. Bright-field images were also captured to observe corresponding morphological changes in the cells following viral infection. **C** Quantification of viral yields comparing SUV39H2 knockdown and NC groups. **D** Expression levels of viral proteins in SCC15 cells with and without SUV39H2 knockdown assessed by western blotting. Tubulin serves as a loading control. **E** Effect of SUV39H2 on oHSV-1 growth. SCC15 cells were infected with oHSV-1 at an MOI of 0.03. Viral particles from cell culture supernatants were collected at designated time points post-infection and quantified by titration on Vero cells. **F** mRNA levels of viral IE gene ICP0, E gene ICP8 and L gene gD in SCC15 cells were measured at 30 hpi with oHSV. Data are expressed as the mean ± SEM. **P < 0.01, ***P < 0.001,****P < 0.0001.

To further validate the role of SUV39H2, we performed knockdown experiments using shRNAs specific to SUV39H2. Western blotting analysis confirmed effective silencing of SUV39H2 in SCC15 cells (Fig. 2A). SUV39H2 knockdown did not significantly affect the proliferation of SCC15 cells (Fig. S4B). SUV39H2-knockdown SCC15 cells (shSUV39H2) and nontarget control (NC) cells were infected with oHSV-1 at MOIs of 0.03 and 0.05 for 30 h, respectively. Infected cells were visualized as EGFP-positive under fluorescence microscopy. SUV39H2 knockdown resulted in a higher percentage of EGFP-positive cells, indicating enhanced viral replication (Fig. 2B). Subsequently, we harvested viral progeny and measured viral titers using a plaque assay. SUV39H2 knockdown significantly increased virus yields of oHSV-1 in SCC15 cells compared to the NC group (Fig. 2C). Additionally, western blotting was performed to assess the expression of the oHSV-1 protein ICP8. SUV39H2 knockdown markedly increased ICP8 expression in SCC15 cells (Fig. 2D). The transcript levels of viral immediate-early (IE) gene *ICP0*, early (E) gene *ICP8* and late (L) gene *gD* were also examined. Knockdown of SUV39H2 increased the levels of viral mRNA in SCC15 cells (Fig. 2F).

To investigate the effect of SUV39H2 on oHSV-1 growth, SUV39H2-depleted SCC15 cells were exposed to 0.03 MOI oHSV-1, and progeny viruses were assayed at various times postinfection. The growth of oHSV-1 in SUV39H2-depleted SCC15 cells was greater than that in infected NC cells. As shown in Fig. 2E, the progeny virus yields in shSUV39H2 cells were 2.7-, 6.1-, and 6.4-fold higher at 36 h, 48 h, and 72 h post-infection (hpi), respectively. These results indicate that knockdown of SUV39H2 enhances viral replication in SCC15 cells, suggesting that SUV39H2 may function as a restricting factor that limits the lytic replication of oHSV-1.

Gain-of-function experiments were conducted to assess the impact of SUV39H2 overexpression on oHSV-1 replication in SCC15 cells. A stable SUV39H2 overexpression cell line was generated, and protein expression levels were confirmed by western blotting (Fig. S1A). The SUV39H2 overexpression (SUV39H2^OE^) and the nontarget control (CON) SCC15 cell lines were infected with oHSV-1 at 0.05 MOI for 30 hpi or 0.1 MOI for 24 hpi, respectively. As shown in Fig. S1B–D, SUV39H2 overexpression reduced the population of EGFP-positive cells, viral yields and the level of viral protein ICP8 expression. The results of viral growth kinetics indicated that progeny virus yields in SUV39H2^OE^ cells were 0.6-, 0.5-, and 0.4-fold lower at 36, 48, and 72 hpi, respectively (Fig. S1E). Taken together, these results suggest that SUV39H2 plays a role in limiting viral replication in SCC15 cells.

### The SUV39H2 inhibitor OTS186935 enhances oHSV-1 replication

**Fig. 3:**
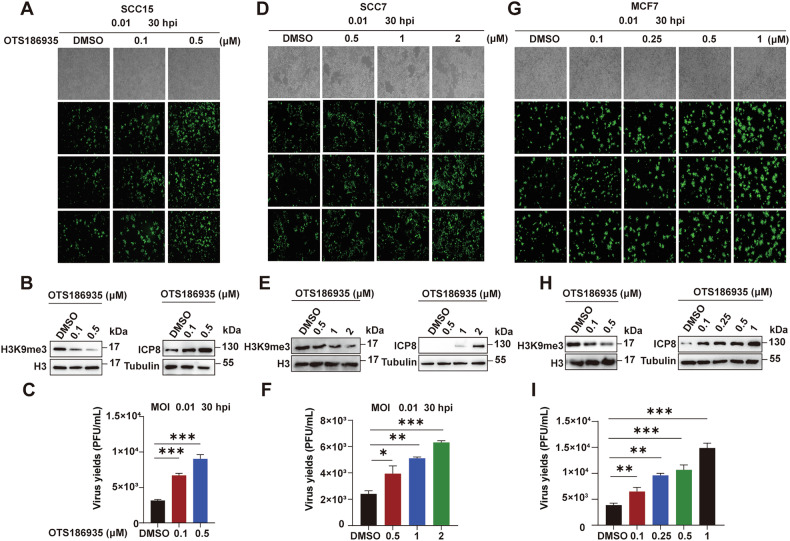
Effects of SUV39H2 inhibitor OTS186935 on viral replication in vitro. **A**, **D**, **G** Fluorescence imaging of viral replication in SCC15, SCC7 and MCF7 cells treated with OTS186935 at different concentrations for 24 h, followed by infection with oHSV-1 at an MOI of 0.01 for 30 h. **B**, **E** and **H**. Western blotting analysis confirmed the inhibitory activity of OTS186935 on SUV39H2 in SCC15, SCC7 and MCF7 cells and demonstrated viral protein expression in both SCC15 and SCC7 cells pre-treated with OTS186935, with tubulin used as the loading control. **C**, **F**, **I** Virus yields in SCC15, SCC7 and MCF7 cells pre-treated with OTS186935. Data are expressed as the mean ± SEM. *P < 0.05, **P < 0.01, ***P < 0.001, ****P < 0.0001.

Our results demonstrate that SUV39H2 functions as a critical restriction factor for oHSV-1 replication in SCC15 cells. To further explore the therapeutic potential of SUV39H2 inhibition, the effects of the SUV39H2 inhibitor OTS186935 on oHSV-1 replication were assessed in SCC15, SCC7and MCF7 cells. Cells were treated with OTS186935 for 24 h, followed by oHSV-1 infection at an MOI of 0.01, resulting in a dose-dependent increase in EGFP-positive cells (Fig. 3A, D, G). Notably, treatment with 0.1 µM and 0.5 µM OTS186935 significantly reduced H3K9me3 levels in SCC15 and MCF7 cells. However, in SCC7 cells, H3K9me3 levels were reduced only at concentrations of 1 µM and 2 µM (Fig. 3B, E, H, left). Pretreatment with OTS186935 in all the three cell lines enhanced the expression of viral proteins (Fig. 3B, E, H, right). Correspondingly, a marked increase in viral yields was achieved in SCC7 cells only at these higher concentrations, with 2.1-fold and 2.6-fold increase in viral yields at 1 µM and 2 µM, respectively. In contrast, SCC15 and MCF7 cells displayed a notable 2-fold increase in viral replication at as low as 0.1 µM. (Fig. 3C, F, I).

Further, we analyzed the effect of SUV39H2 on T-VEC replication. T-VEC was engineered using CRISPR/Cas9 and confirmed to express GM-CSF (Fig. S2A, B). SCC15 cells pretreated with OTS186935 and subsequently infected with T-VEC exhibited comparable outcomes, showing a dose-dependent increase in viral yields and viral proteins (Fig. S3A). Additionally, treatment with 0-3 µM OTS186935 had no significant effect on the proliferation of SCC15, SCC7, and MCF7 cells, with cell viability remaining above 90% (Fig. S4A). In summary, inhibition of SUV39H2 by OTS186935 reduced H3K9me3-mediated transcriptional repression at viral promoters, promoting viral replication and increasing cellular susceptibility to oHSV-1 induced oncolysis.

### Combined OTS186935 and oHSV-1 treatment exhibits potent anti-tumor efficacy in SCC15 tumors

**Fig. 4:**
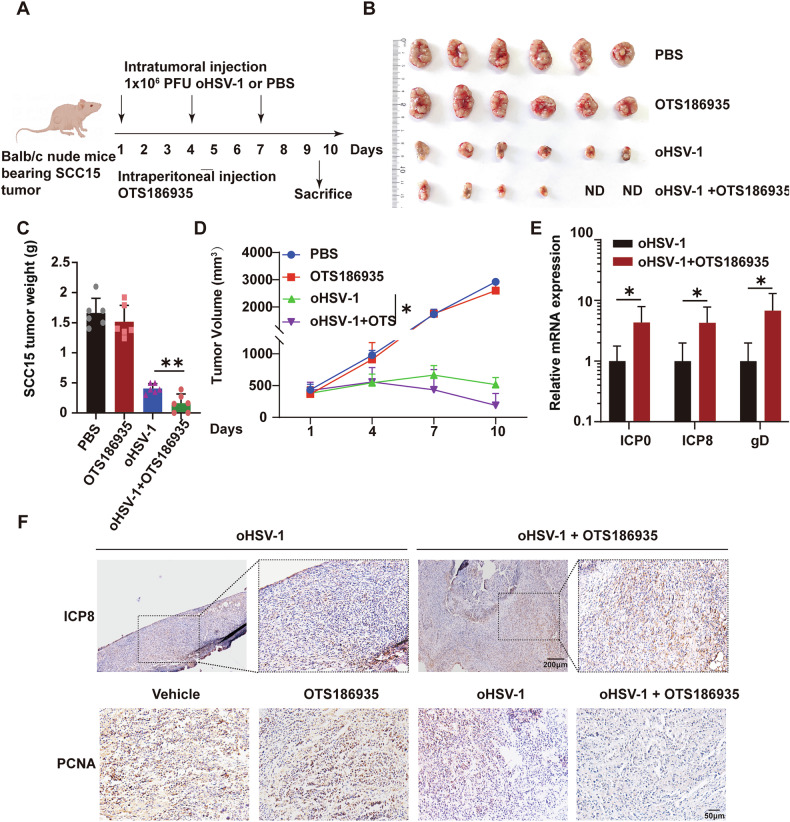
The anti-tumor efficacy of combined treatment of oHSV-1 and OTS186935 in SCC15 tumors. **A** The detailed description of the mouse study design is provided in the main text. **B** Mice were treated with PBS, OTS186935, oHSV-1, or a combination of oHSV-1 and OTS186935. On day 10 following the initial treatment, tumors were excised and photographed. ND: no detected. **C** Tumor weights were also measured on day 10. **D** Tumor volume was measured every two days for a consecutive 10 days and calculated using the formula: length × width² × 0.5. **E** Examination of viral mRNA levels in SCC15 tumors, *n* = 5. **F** IHC was employed to evaluate the levels of viral protein ICP8 in sections of xenografts tumor. Scale bar, 200 μm. Images of PCNA-positive cells (PCNA: a marker of cell proliferation). Scale bar, 50 μm. Data are expressed as the mean ± SEM. *P < 0.05, **P < 0.01.

We investigated the anti-tumor effects of combining OTS186935 with oHSV-1 in SCC15 tumor models. As shown in Fig. 4A, SCC15 cells (1 × 10^6^) were injected subcutaneously into nude BALB/c mice. Once the tumor volume reached 100-200 mm³, mice were randomized into four treatment groups (*n* = 6) to initiate treatment. Mice received daily intraperitoneal injections of 5 mg/kg OTS186935 or vehicle control (1% DMSO in PBS). Intratumoral injections of 1 × 10^6^ PFU of oHSV-1 (50 μL) were administered on days 1, 4, and 7. Every three days over a 10-day period, tumor dimensions were measured in length and width. On day 10 post-treatment, all mice were sacrificed, and the tumors were excised and weighed. As shown in Fig. 4B, C, tumor size and weight were significantly reduced in the combined treatment group compared to the DMSO and monotherapy groups. Notably, in the combination treatment group, two mice were completely cured. The average tumor volumes for SCC15 tumors across different treatment groups were calculated and compared. As depicted in Fig. 4D, the tumors growth in combination treatment group decreased significantly. qRT-PCR analysis showed that OTS186935 treatment increased the accumulation of viral ICP0, ICP8, and gD mRNA in SCC15 tumors (Fig. 4E). Additionally, IHC staining showed increased expression of viral protein ICP8 and reduced cellular proliferation in the combination treatment group, indicating enhanced viral replication and oncolytic activity (Fig. 4F). Overall, these results demonstrate that OTS186935 enhances the anti-tumor efficacy of oHSV-1 by promoting viral replication, leading to improved tumor suppression in SCC15 models, indicating that the suppression of SUV39H2 facilitates viral infection establishment and represents a promising therapeutic strategy.

### SUV39H2 represses oHSV-1 gene transcription by catalyzing H3K9me3 at viral promoters

**Fig. 5:**
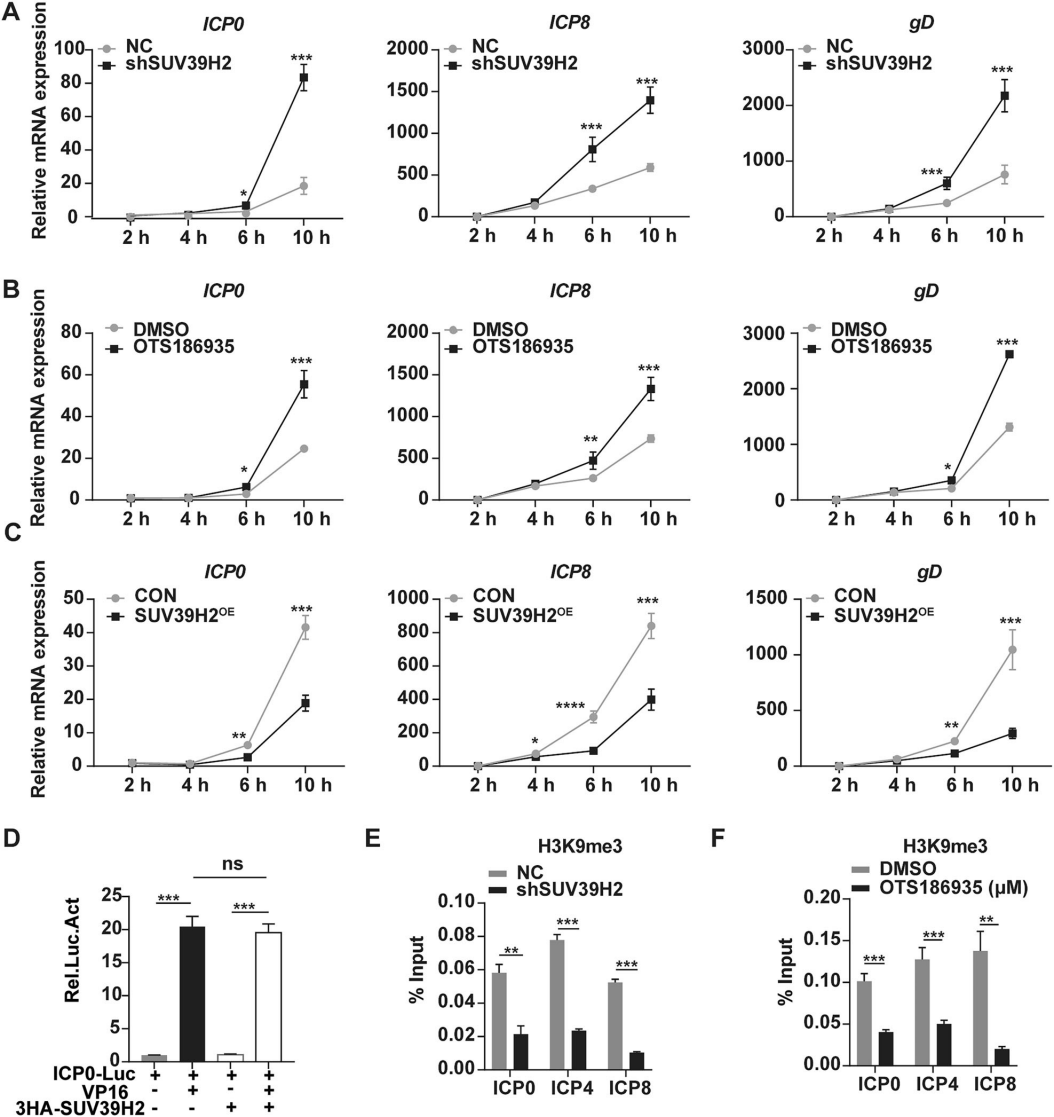
SUV39H2 inhibits oHSV-1 gene transcription by binding to the viral promoter. **A**–**C** SCC15 cells with either SUV39H2 knockdown or overexpression, or those treated with OTS186935, were infected with oHSV-1 at an MOI of 1, and samples were collected at various time points post-infection. The transcriptional levels of IE (*ICP0*), E (*ICP8*), and L (*gD*) viral genes were quantified by qRT-PCR. **D** HEK293T cells were co-transfected with VP16 (25 ng) and ICP0-Luc (10 ng), combined with 3HA-SUV39H2 or empty vector. At the same time, pCMV-β-gal (50 ng) was transfected to normalize the transfection efficiency. At 24 h post-transfection, luciferase activities were measured and corrected by b-gal catalytic activities. **E** SUV39H2 knockdown SCC15 cells and NC cells infected with oHSV-1 (MOI = 3) for 3 h. The ChIP assay shown the levels of H3K9me3 on viral promoters. **F** SCC15 cells treated with 0.5 μM OTS186935 for 24 h and then infected with oHSV-1 (MOI = 3) for 3 h. The ChIP assay shown the levels of H3K9me3 on viral promoters. Data are expressed as the mean ± SEM. *P < 0.05, **P < 0.01, ***P < 0.001, ****P < 0.0001.

SUV39H2 is a histone methyltransferase that catalyzes H3K9me3, a repressive chromatin modification associated with transcriptional silencing. Additionally, SUV39H2 interacts with non-histone proteins, suggesting broader regulatory roles. We hypothesized that SUV39H2 could influence the transcription of viral genes. To investigate this, we first analyzed the temporal cascade of oHSV-1 gene transcription. SUV39H2 knockdown significantly increased the transcription of the immediate-early (IE) gene ICP0, the early (E) gene ICP8, and the late (L) gene gD as early as 6 (hpi) (Fig. 5A). A similar increase in viral gene transcription was observed in SCC15 cells treated with 0.5 µM of the SUV39H2 inhibitor OTS186935 (Fig. 5B). Conversely, overexpression of SUV39H2 resulted in the suppression of viral transcription levels (Fig. 5C).

VP16 (Viral Protein 16) is a key virion-associated protein that initiates the sequential expression of HSV genes by activating IE gene transcription. To determine whether SUV39H2 influences VP16-mediated transactivation, HEK293T cells were co-transfected with ICP0-Luc, VP16, and 3HA-SUV39H2 and incubated for 24 h. While VP16 robustly activated ICP0 transcription, SUV39H2 had no significant effect on VP16-driven transactivation (Fig. 5D). To further investigate the mechanism by which SUV39H2 regulates oHSV-1 gene transcription, chromatin immunoprecipitation (Chip) assays were performed to assess H3K9me3 levels at oHSV-1 promoters. Chip analysis revealed a reduction in H3K9me3 marks at the promoters of oHSV-1 IE (ICP0, ICP4) and E (ICP8) genes in SUV39H2 knockdown cells, suggesting decreased transcriptional repression (Fig. 5E). Similar outcomes were observed in SCC15 cells pretreated with OTS186935 prior to infection with oHSV-1 (Fig. 5F). These results suggest that SUV39H2 represses oHSV-1 gene transcription by catalyzing H3K9me3 at viral promoters, thus limiting viral replication.

### oHSV-1 infection induces SUV39H2 degradation via the ubiquitin-proteasome pathway

**Fig. 6:**
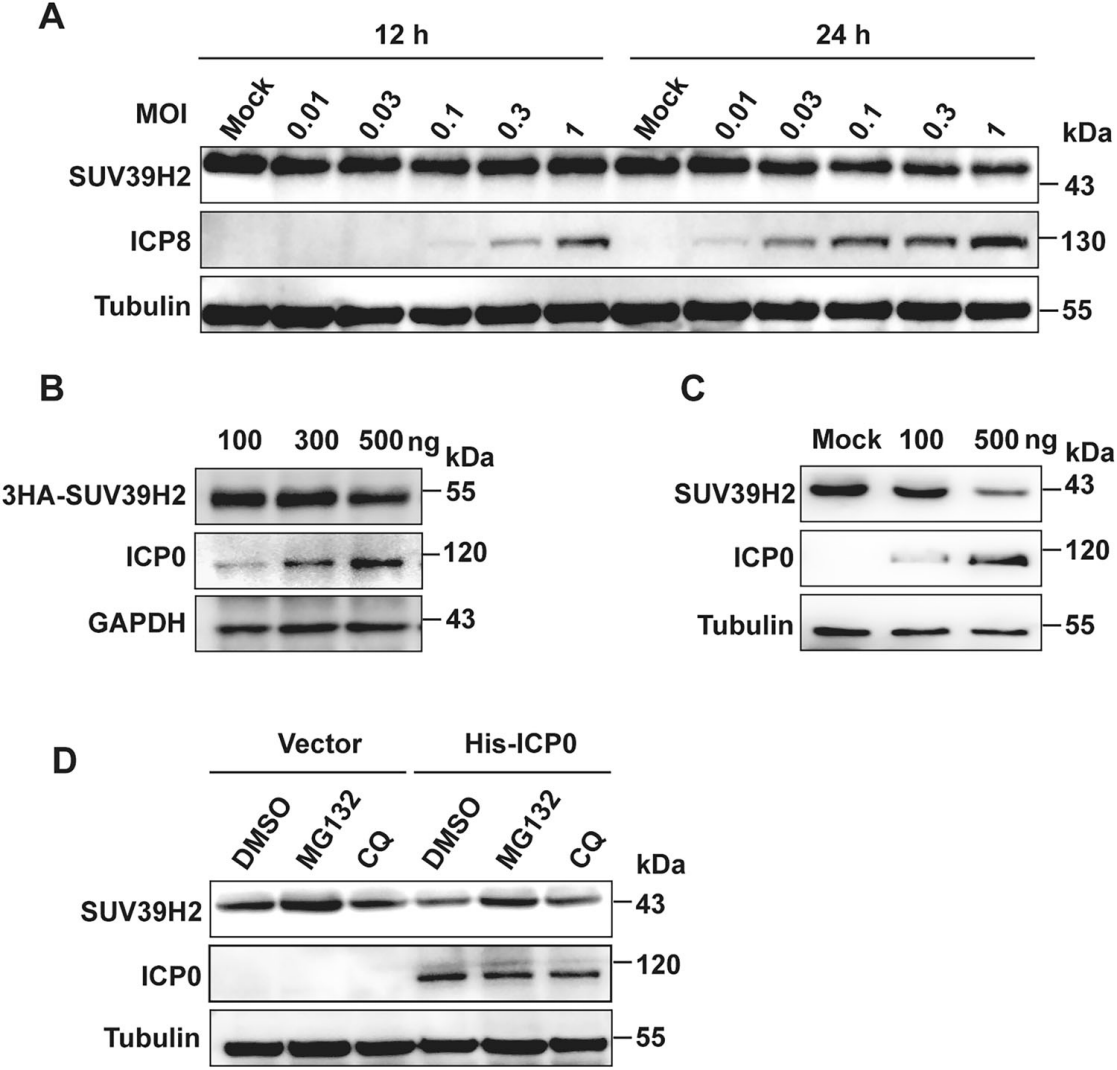
oHSV-1 induces SUV39H2 degradation via the ubiquitin-proteasome pathway in an ICP0-dependent manner. **A** SCC15 cells were infected with oHSV-1 at increasing multiplicities of infection (MOI, 0.01–1). Western blot analysis was performed to assess SUV39H2 protein levels at 12 and 24 h post-infection (hpi). Tubulin was used as a loading control. **B** SCC15 cells were transfected with increasing amounts of His-ICP0 plasmid (100–500 ng) for 48 h. Western blot analysis revealed a dose-dependent decrease in endogenous SUV39H2 protein levels. Tubulin was used as a loading control. **C** HEK293T cells were co-transfected with 3HA-SUV39H2 and increasing amounts of His-ICP0 (100–500 ng). Western blot analysis demonstrated a dose-dependent reduction in 3HA-SUV39H2 protein levels. GAPDH was used as a loading control. **D** SCC15 cells were transfected with His-ICP0 (500 ng) for 40 h, followed by treatment with MG132 (10 μM, proteasome inhibitor) or chloroquine (CQ, 50 μM, lysosomal inhibitor) for 3 h. Western blot analysis showed that MG132 restored SUV39H2 levels, while CQ had no effect, indicating that ICP0 promotes SUV39H2 degradation via the ubiquitin-proteasome pathway. β-Tubulin was used as a loading control.

To elucidate the impact of oncolytic herpes simplex virus-1 (oHSV-1) infection on SUV39H2 protein stability, SCC15 cells were exposed to varying multiplicities of infection (MOI) ranging from 0.01 to 1. Western blotting revealed no significant alterations in SUV39H2 protein abundance at 12 hpi across all MOI conditions, suggesting that the early phase of infection does not trigger its immediate degradation. However, by 24 hpi, a pronounced reduction in SUV39H2 abundance was detected in cells infected at 0.1 MOI, with degradation efficiency intensifying at higher MOI values (0.3–1), indicating a dose- and time-dependent proteolytic regulation of SUV39H2 (Fig. 6A).

Given that ICP0 functions as an E3 ubiquitin ligase, it is likely to mediate the degradation of SUV39H2. To investigate this, SCC15 cells were transfected with increasing amounts of His-ICP0 plasmids (100 ng, 500 ng), and western blotting analysis revealed a dose-dependent decrease in endogenous SUV39H2 (Fig. 6B). Similar results were observed in HEK293T cells co-transfected with 3HA-SUV39H2 and His-ICP0 (100 ng, 300 ng, 500 ng), further corroborating ICP0-mediated SUV39H2 depletion (Fig. 6C).

To investigate the degradation pathway of SUV39H2, inhibitor treatment assays were performed. SCC15 cells were transfected with His-ICP0 for 40 h, followed by treatment with 10 μM MG132 (a proteasome inhibitor) or 50 μM chloroquine (CQ, a lysosomal inhibitor) for 3 h. Western blotting analysis revealed that MG132 markedly restored SUV39H2 levels in control cells, whereas CQ had no discernible effect, indicating that SUV39H2 undergoes basal degradation via the proteasome. Notably, in the presence of MG132, ICP0-mediated SUV39H2 degradation was effectively blocked, confirming that SUV39H2 is targeted for proteasomal degradation in an ICP0-dependent manner (Fig. 6D). These findings establish a mechanistic link between oHSV-1 infection and SUV39H2 turnover via the ubiquitin-proteasome pathway, highlighting a novel regulatory axis in viral-host interactions.

### Depletion of SUV39H2 enhances the oncolytic effects of oHSV-1 in SCC7 cells and tumors

**Fig. 7:**
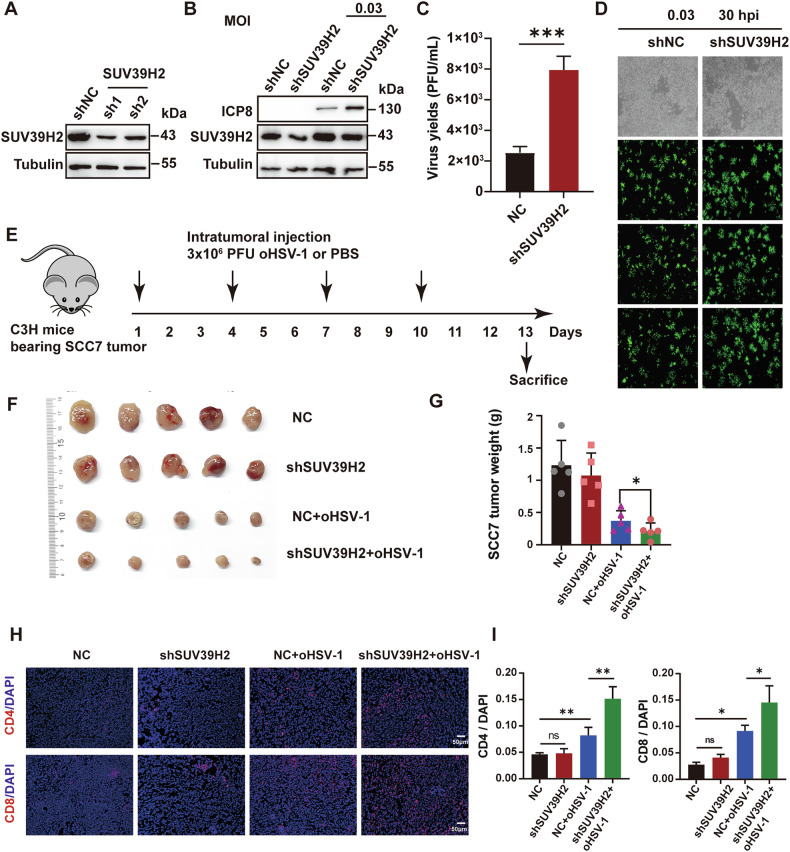
Enhanced oncolytic effects of oHSV-1 in SCC7 cells and tumors following SUV39H2 Depletion. **A** Western blotting analysis confirming the efficiency of SUV39H2 knockdown in SCC7 cells following shRNA treatment, with tubulin used as a loading control. **B** Examination of viral protein levels in SUV39H2-knockdown SCC7 cells infected with oHSV-1 at an MOI of 0.03 for 30 h. **C** Virus yields in SUV39H2-knockdown SCC7 cells infected with oHSV-1 at an MOI of 0.03 for 30 h. **D** Fluorescence imaging of SCC7 SUV39H2 knockdown cells infected with oHSV-1 at an MOI of 0.03 for 30 h to assess viral replication. **E** The detailed description of the mouse study design is provided in the main text. **F**, **G** SCC7 knockdown cells were implanted into mice and treated with oHSV-1. On day 13 following the initial treatment, mice were sacrifice, and tumors were excised, photographed, and weighed to evaluate tumor burden. **H** Images of CD4 and CD8 positive areas in tumor tissues assessed by IF. Scale bar, 100 μm. **I** Quantification of CD4 and CD8-positive areas in (**H**). Data are expressed as the mean ± SEM. *P < 0.05, **P < 0.01, ***P < 0.001.

To evaluate the efficacy of combination therapy in an immunocompetent environment, we conducted experiments using SCC7 cells in immunocompetent C3H/HeN mice. Due to the high concentration of OTS186935 required for SCC7 cells, we opted to investigate the effects of SUV39H2 knockdown on oHSV-1 replication and oncolytic efficacy instead. Lentiviral transduction of SUV39H2-specific shRNAs efficiently suppressed SUV39H2 expression (Fig. 7A). The most efficient shRNA (shRNA1) was selected for subsequent experiments. Protein levels of ICP8 were upregulated following SUV39H2 depletion in SCC7 cells (Fig. 7B). It was demonstrated that SUV39H2 knockdown significantly enhanced oHSV-1 replication, as evidenced by fluorescence assays and viral yields (Fig. 7C, D). These results indicate that SUV39H2 depletion in SCC7 cells enhance viral replication.

OSCC allografts were subsequently established in C3H/HeN mice using SUV39H2 knockdown cells and scrambled control cells. Consistent with the above, once tumors reached a size of 100-200 mm^3^, a treatment of 50 μL oHSV-1 (3 × 10^6^ PFU) in PBS was administered. On day 13 post-treatment, the mice were sacrificed and the samples were harvested (Fig. 7E). As Fig. 7F, G, depletion of SUV39H2 combined with oHSV-1 treatment markedly improved oncolytic activity, reducing tumor weight. Furthermore, immunofluorescence quantification demonstrated a 1.5-fold increase in CD8+ T cell density and 1.7-fold increase in CD4+ T cells within SUV39H2-depleted tumors compared to controls (Fig. 7H, I), indicating enhanced T cell infiltration upon combination therapy. Overall, these findings suggest that SUV39H2 knockdown enhances oHSV-1 replication, improves therapeutic efficacy in SCC7 tumors, and promotes a more robust antitumor immune response, supporting its potential as a target in oncolytic virotherapy.

### SUV39H2 expression correlates with immune infiltration and checkpoint expression in cancers

**Fig. 8:**
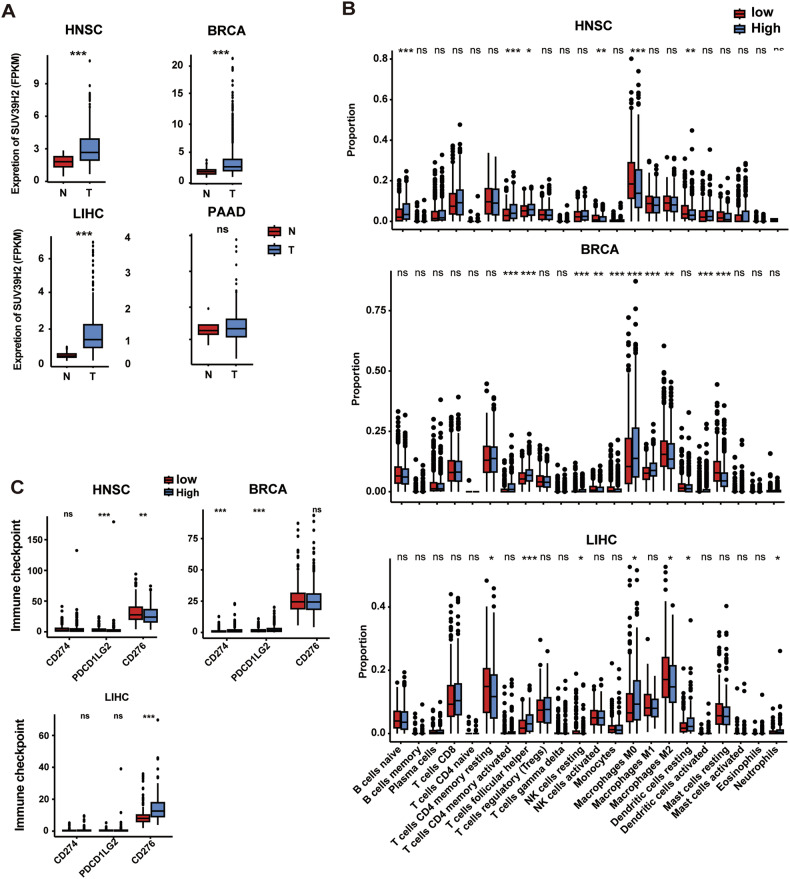
Correlation of SUV39H2 expression with immune cell infiltration and IC expression in four cancer types. **A** Expression of SUV39H2 between tumor tissues (T) and normal tissues (N). **B** Changes of immune microenvironment with different expression of SUV39H2. **C** Expression of IC molecules in tissues with high and low SUV39H2 expression.

To explore the possible immune-regulatory role of SUV39H2 in different cancer types, we analyzed the relationship between SUV39H2 expression, immune cell infiltration, and immune checkpoint molecule expression in HNSCC, LIHC (Liver Hepatocellular Carcinoma), BRCA (Breast Invasive Carcinoma), and PAAD (Pancreatic Adenocarcinoma) tumors based on data from the TCGA database. SUV39H2 expression was higher in HNSCC, LIHC, and BRCA tumor tissues compared to normal tissues, with no significant difference observed in PAAD tumors (Fig. 8A). Immune infiltration analysis showed associations between SUV39H2 expression and various immune cells, such as B cells, T cells, NK cells, and macrophages. SUV39H2 appears to differentially impact immune cell infiltration across tumor types (Fig. 8B). Based on the median expression of SUV39H2, tumor samples were classified into high-expression and low-expression groups. Compared to the low-expression group, HNSCC high-expression samples showed reduced PDCD1LG2 and CD276 expression, while BRCA high-expression samples showed decreased CD274 and PDCD1LG2 levels. In LIHC, high SUV39H2 expression was associated with increased CD276 levels. These findings suggest that SUV39H2 not only potentiates viral replication but also modulates immune check (IC) point networks, which may collectively dictate patient responsiveness to oncolytic virotherapy (Fig. 8C). Overall, these results indicate that SUV39H2 may serve as a potential biomarker to predict the effectiveness of oncolytic virus therapy.

## Discussion

Oncolytic virus strategies have emerged in recent decades as promising alternatives to radiotherapy and chemotherapy for cancer treatment. Although past and current clinical trials have demonstrated the safety and limited efficacy of oHSV-1, several critical challenges remain to be overcome. In this study, through Genome-wide CRISPR/Cas9 screening identified SUV39H2 as a key gene mediating oHSV-1 resistance in OSCC. SUV39H2 inhibits oHSV-1 replication by catalyzing H3K9me3 at the viral promoter, thereby suppressing viral gene transcription. Additionally, inhibition of SUV39H2 significantly enhanced the oncolytic effect of oHSV-1 in OSCC.

The identification of SUV39H2 as a critical factor in oHSV-1 resistance suggests several potential therapeutic strategies. One possible approach involves combining oHSV-1 therapy with inhibitors of SUV39H2 or other chromatin-modifying enzymes to enhance viral replication. OTS186935, a potent inhibitor of SUV39H2, significantly suppressed tumor growth in mouse xenograft models with MDA-MB-231 breast cancer, HGC-27 gastric cancer, and A549 lung cancer cell lines. Notably, this compound achieved significant antitumor efficacy without observable toxicity in treated subjects [38, 39]. Our study showed that OTS186935 pretreatment effectively suppressed SUV39H2 activity in SCC15, SCC7 and MCF7 cells, significantly enhancing oHSV-1 replication. Interestingly, 0.5 µM OTS186935 enhanced oHSV-1 replication in SCC15 and MCF7 cells, whereas higher concentrations were needed in SCC7 cells, likely reflecting the structural differences between human and mouse SUV39H2. Combination therapy in SCC15 xenograft models exhibited enhanced antitumor efficacy relative to oHSV-1 monotherapy. These highlight the potential therapeutic advantage of combining SUV39H2 inhibitors with oHSV-1 to enhance oncolytic efficacy while minimizing toxicity. An alternative approach is to construct a recombinant oncolytic virus carrying shRNA targeting SUV39H2. Various oncolytic viruses carrying shRNA have been reported to effectively enhance therapeutic outcomes, for example, oncolytic adenoviruses encoding shRNA effectively downregulate target proteins such as DNA-PKcs, Apollon, and VEGF, enhancing radiation sensitivity, antitumor effects of 5-fluorouracil, and inhibiting angiogenesis and tumor growth in various cancer models [40–42]. Future research should explore whether the construction of shRNA-encoding viruses can effectively optimize therapeutic outcomes, providing a pathway to refine oncolytic therapy with enhanced specificity and safety.

H3K9me3 (a hallmark of constitutive heterochromatin in eukaryotes), may inhibits viral gene expression by altering chromatin states surrounding the viral genome. Previous studies have indicated that repressive modifications, including H3K9me3 create a transcriptionally repressive environment that inhibits the replication of various DNA viruses, including human papillomavirus and Epstein-Barr virus [43, 44]. HSV-1 replication is regulated by histone modifications, which play a key role in controlling viral gene transcription and replication. Although previous studies have shown that EZH2/1 inhibitors significantly downregulate HSV-1 immediate-early (IE) gene expression by inhibiting histone H3K27 methylation [45], and that IFI16 can inhibit viral replication by remodeling H3K9me3/H3K4me3 epigenetic marks [46], there is still limited understanding of the specific network of methyltransferases that regulate the HSV life cycle. The virus-specific recruitment of methyltransferases and their spatiotemporal regulatory mechanisms remain critical areas for further investigation. Our data demonstrate that depletion of SUV39H2 reduces H3K9me3 levels at viral promoter regions resulting in increased viral gene expression. These data support that SUV39H2 is a functional methyltransferase regulating HSV-1 gene expression. SUV39H1 and SUV39H2 were the first protein lysine methyltransferases discovered over two decades ago. Both enzymes catalyze di- and trimethylation of H3K9, playing crucial roles in heterochromatin maintenance and gene silencing. Their prognostic significance varies across cancer types, with SUV39H1 expression showing context-dependent risk, while SUV39H2 high expression typically indicates an unfavorable prognosis [47]. Interestingly, in herpes simplex virus 1 (HSV-1) ICP0-mutant infections, SUV39H1 knockdown slightly reduced viral yield. Chaetocin-mediated SUV39H1 inhibition in human foreskin fibroblasts (HFFs) decreased viral productivity [48]. In hepatitis B virus (HBV), SUV39H1, which degrades Cdt2, thereby enhances viral loads [49]. The differential effects of SUV39H1 and SUV39H2 on viral replication may stem from SUV39H1’s preferential targeting of host factors rather than viral genomic elements.

Our study reveals that oHSV-1 infection leads to the degradation of SUV39H2 in a dose- and time-dependent manner, with significant reduction observed at 24 hpi at higher viral titers. Mechanistic investigation reveals that this degradation is mediated by the viral E3 ubiquitin ligase ICP0 through the ubiquitin-proteasome system, as evidenced by proteasome inhibitor rescue experiments. Given SUV39H2’s established role in maintaining transcriptional repression via H3K9me3-mediated heterochromatin formation, its virus-triggered degradation likely represents an evolved viral tactic to dismantle host epigenetic defenses, thereby liberating viral promoters (e.g., ICP0, ICP4, and ICP8) from chromatin silencing and facilitating viral gene transcription. This highlights the intricate interplay between oHSV-1 and host epigenetic regulation. Animal experiments demonstrated that preemptive administration of the inhibitor significantly enhanced therapeutic efficacy, highlighting the crucial role of chromatin silencing in the early post-entry phase of viral infection and underscoring the necessity of targeted intervention at this critical stage.

While our study demonstrates that SUV39H2 influences oHSV-1 replication and oncolytic efficacy, several limitations should be acknowledged. Our CRISPR screen identified SUV39H2 as a key resistance factor in OSCC, additional in vivo validation using orthotopic or metastatic models is needed to confirm its relevance in more clinically representative contexts. The use of cell lines and xenograft models, while informative, may not fully capture the complexity of the tumor microenvironment, especially immune components that interact with oncolytic viruses. Finally, while combination therapy with oHSV-1 and SUV39H2 inhibitors showed promising results, future studies should evaluate long-term toxicity, viral persistence, and the feasibility of integrating additional immunotherapies.

In conclusion, by using genome-wide CRISPR/Cas9 library screening, we identified SUV39H2 as a critical gene suppressed oHSV-1 replication in OSCC. Combining SUV39H2 inhibition with oHSV-1 enhanced oncolytic efficacy both in xenograft and syngeneic tumor models. This finding underscores the importance of chromatin modifications in regulating oHSV-1 replication and offers a promising target for improving oncolytic virotherapy efficacy.

## Materials and methods

### Genome-wide CRISPR/Cas9 knockout library screen

SCC15 cells were transduced with the GeCKO v2 libraries A and B (The Human GeCKOv2A CRISPR knockout pooled library [50], Addgene # 1000000049), targeting 19,050 human coding genes with three sgRNAs per gene and 1864 miRNAs (four sgRNAs per miRNA, and 1000 non-targeting controls) at an MOI of 0.3 to ensure effective barcoding of individual cells. The cells were subsequently selected using 2 μg/mL puromycin and cultured for seven days to generate a mutant cell pool. At least 6 × 10^7^ cells were collected as baseline (estimated coverage: 500 cells/sgRNA). For the genome-wide CRISPR screening assays, mutant cells were infected with oHSV-1 at an MOI of 0.01 for 48 and 72 h, respectively. After treatment, at least 6 × 10^7^ cells were collected for genomic DNA extraction, and the abundance of sgRNAs was determined by GENEWIZ (Tianjin, China) using the MAGeCK algorithm.

### Cells, animals and reagents

The HEK293FT-sgEGFP cell line was stably transduced by lentiviruses with lentiviruses containing two lentiCRISPR-sgRNA plasmids targeting the EGFP-coding sequence. The OSCC lines SCC15 and SCC7 were obtained from the FengHui Biotechnology (China). Vero, HEK293T and MCF7 cell lines are maintained in our laboratory. All cell lines were cultured in DMEM high glucose (Thermo Scientific, USA) supplemented with 10% fetal bovine serum (FBS; Thermo Scientific, USA). OTS186935 (HY-122181) and SCR7 (HY-12742) were purchased from MedChem Express (MCE, USA). All animals in this study were obtained from Vital River Laboratories (Beijing, China).

### Generation of stably transduced cell lines

The shRNA sequences targeting human SUV39H2 (shRNA1: 5′-CCAAATCTTCAGGTGTTCAAT-3′; shRNA2: 5′-CTCTAATGACAAGCATAATTA-3′) and mouse SUV39H2 (shRNA1: 5′-CGGTAGATATTTGGTGGTTAA-3’; shRNA2: 5′-CGGGGCACATAAACGGTAGATAT-3’) were cloned into the pSIREN-RetroQ vector. HEK293T cells were transfected with 3 μg of pMLV-Gag-Pol, 2 μg of pVSV-G, and 5 μg of pSIREN-RetroQ (with shRNA cloned) for 48 h. Subsequently, the lentiviruses were utilized to infect SCC15 or SCC7 cells. After 48 h of infection, the cells were incubated with 2 μg/mL puromycin for 7 days. To establish cell lines stably expressing SUV39H2, the coding region was amplified and inserted into the pQCXIP vector. The plasmid was co-transfected with pMLV-Gag-Pol, pVSV-G, and pQC-SUV39H2 for 48 h to produce lentiviruses. Subsequently, the lentiviruses were used to infect the target cells for 48 h, after which the cells were incubated with 2 μg/mL puromycin for 7 days.

### Viruses

oHSV-1 was generated from the wild-type HSV-1 (F strain) backbone, in which both copies of the ICP34.5 coding region were substituted with the EGFP gene, and the ICP47 gene was deleted. T-VEC was constructed from oHSV-1 by CRISPR/Cas9-based gene editing. The coding sequence of GM-CSF with the homology arms of genomic regions near the ICP34.5-coding sequences was subcloned into pCDH plasmid and transfected into HEK293FT-sgEGFP cells. At 24 h post transfection, infected with oHSV-1 at an MOI of 0.1 and cultured in the presence of 10 μM SCR7 for 24 h. The harvested viruses were used to infect Vero cells, and the EGFP-negative plaques were picked under fluorescence microscopy. Viruses from single plaque were amplified with GM-CSF primers (F: 5′-ATGTGGCTGCAGAGCCTG-3′, R: 5′-TTACTCCTGCACTGGCTCCC-3′) and the process was repeated for five rounds. The last round plaque-purified viral particles were verified by PCR and ELISA for GM-CSF (Fig. S2).

### Virus propagation and purification

Vero cells were infected with virus. When 100% cytopathic effect (CPE) was observed, cells were collected and lysed through three freeze-and-thaw cycles. The supernatant was centrifuged and collected; an equal volume of 9% sterile milk was added, aliquoted, and stored at −80 °C. The virus for in vivo injection was purified. Briefly, Vero cells were infected with the indicated viruses at an MOI of 0.1 for 48 h. The supernatant went through the HiScreen Capto Core 700 (17-5481-15; GE Healthcare, USA) with an ÄKTA protein purification system to obtain the purified viruses. The purified viruses were concentrated using an ultrafiltration tube (UFC910096; Millipore, USA) and stored at −80 °C.

### Virus titration assay

1 × 10^5^ Vero cells were seeded in 24-well plates. The virus or supernatants were serially diluted and used to infect the Vero cells for 1 h. The cells were washed twice with PBS and then covered with DMEM high glucose containing 2% FBS. At 36 hpi, plaques in parallel wells were counted in triplicate, and the mean value was calculated. Virus titers were presented as plaque forming unit (PFU)/mL.

### Plasmid transfection

SCC15 and HEK293T cells were seeded in 12-well plates at a density of 0.25 × 10^6^ cells per well and cultured overnight to achieve approximately 70–80% confluence. Plasmid transfection was performed using Lipofectamine 3000 (Invitrogen, USA) according to the manufacturer’s instructions. Briefly, plasmid DNA was diluted in Opti-MEM (Gibco, USA) and mixed with Lipofectamine 3000 reagent. After a 15-min incubation at room temperature, the transfection mixture was added dropwise to the cells. Following a 6-h incubation, the culture medium was replaced with fresh complete medium.

### qRT-PCR

Total RNA was extracted using TRIzol reagent (Invitrogen, USA). Next, 1 μg total RNAs were reverse transcribed to cDNA using the HiScript II 1st Strand cDNA Synthesis Kit (R212-01, Vazyme, China). mRNA accumulation was analyzed by quantitative PCR using ChamQ Blue Universal SYBR qPCR Master Mix (Q312-02, Vazyme, China) in Step on plus Real-time PCR system (Applied Biosystems, USA). *GAPDH* was used as the normalization control. All assays were independently performed in triplicate. Relative quantity of gene expression was determined with the 2-ΔΔCT method. The sequences of PCR primers, synthesized by GENEWIZ (Tianjin, China), were as follows: Human *GAPDH* primer (F: 5′-AACAGCGACACCCACTCCTC-3′, R: 5′-CATACCAGGAAATGAGCTTGACAA-3′); Mouse *GAPDH* primer (F: 5′-CATGGCCTTCCGTGTTCCTA-3′, R: 5′-CCTGCTTCACCACCTTCTTGAT-3′); *ICP0* primer (F: 5′-CCCACTATCAGGTACACCAGCTT-3′, R: 5′-CTGCGCTGCGACACCTT -3′); *ICP8* primer (F: 5′-CCCAGCACCCAGGCCCCGAACC-3′, R: 5′-AGCGCCTCCCCCGTCGTCTCGT -3′); *gD* primer (F: 5′-CTATGACAGCTTCAGCGCCGTCAG-3′, R: 5′-CGTCCAGTCGTTTATCTTCACGAGC-3′); Human *SUV39H2* primer (F: 5′-AGGCAGGGACCTTGTATATTCC-3′, R: 5′-TGTACTCGGCCAGTGTATCTC-3′); Mouse *SUV39H2* primer (F: 5′-CTAAGAGTTCCCCCGGCTCA-3′, R: 5′-CTCTCAAACCAGGCCAAGAGT-3′).

### Western blotting

Harvested cells were lysed in radioimmunoprecipitation assay (RIPA) buffer supplemented with 1×EDTA-free protease inhibitor cocktail (Roche, Switzerland) on ice for at least 30 min. The supernatants were heat-denatured in 1× loading buffer and separated by SDS-PAGE, transferred to polyvinylidene fluoride (PVDF) membranes (Millipore, USA) and detected with indicated antibodies. Antibodies used in this study included the following: anti-Human-SUV39H2 (A3705, ABclonal, China), anti-Mouse-SUV39H2 (A5855, ABclonal, China), anti-Tubulin (sc-8035, Santa Cruz, USA), anti-ICP8 (sc-53329, Santa Cruz, USA), anti-ICP0 (sc-53070, Santa Cruz, USA), anti-HA(AE008, ABclonal, China), anti-H3 (9715, Cell Signaling Technology, USA), and anti-H3K9me3 (13969, Cell Signaling Technology, USA).

### ELISA

293 T cells were transfected with 0.5 μg pCDH-GM-CSF or infected with T-VEC virus at an MOI of 0.1. After 48 h, the supernatant was collected, and GM-CSF levels in the cell culture supernatant were quantified using ELISA according to the manufacturer’s instructions. ELISA kits were obtained from Solarbio (SEKH-0057).

### Luciferase reporter assays

HEK293T cells in 12-well plates were transfected with 3HA-VP16, 3HA-SUV39H2 and the ICP4-luc along with β-gal. The cells were harvested 24 h post-transfection. Luciferase activity was quantified using the luciferase reporter assay system kit from Promega (Madison, WI, USA) following the manufacturer’s protocol. β-gal activity served as an internal control to normalize transfection efficiency. All experiments were conducted in triplicate and repeated at least three times.

### CCK8 assay

Cells seeded in 96-well plates at 37 °C for overnight incubation were treated with 2% DMEM high glucose in the presence of different concentrations of OTS186935 for 24 h. Cell viability was assessed using the CCK8 Assay Kit (C0042, Beyotime) over 1 to 4 h at 37 °C, and the optical density at 450 nm (OD450) was measured using a BioTek Epoch (BioTek Instruments, USA).

### Chromatin immunoprecipitation (Chip) assay

SCC15 cells were infected with oHSV-1 at an MOI of 5. At 3 hpi, cells were washed 3 times with ice-cold PBS and then scraped from culture dishes into microcentrifuge tubes. The cells were collected by centrifugation at 3000 × *g* at 4 °C for 5 min. The cells were lysed in lysis buffer with 1×EDTA-free protease inhibitor cocktail and sonicated to obtain DNA fragments ranging from 200 bp to 500 bp in length. The samples were clarified by centrifugation at 13,000 × *g* at 4 °C for 10 min. 10 µl from each chromatin supernatant was reserved as the input sample, while 100 µl was incubated with IgG antibody as a negative control, and another 100 µl was incubated with 5 μg antibody specific for H3K9me3 overnight at 4 °C with rotation. Protein A/G beads (B23201, Selleck, China) were used for immunoprecipitation. After 2 h at 4 °C with rotation, the beads were washed at 4 °C with rotation for 5 min, twice with low-salt buffer, once with high-salt buffer, once with LiCl buffer, and twice with TE buffer. Immunocomplexes were eluted by adding 250 μL of elution buffer and incubating for 30 min at 65 °C, followed by collection of the supernatant using a magnetic rack. Cross-links were reversed by incubating for 5 h at 65 °C with a final concentration of 200 mM NaCl. The samples were subsequently treated with RNase A and digested with proteinase K. DNA was purified using a PCR Purification Kit (DC301, Vazyme, China). Immunoprecipitated DNA was detected via qRT-PCR using primers specific for the *ICP0*, *ICP4*, and *ICP8* promoters.

### Animal test

Five-week-old female BALB/c nude mice were subcutaneously injected with 1 × 10^6^ SCC15 cells, and tumor-bearing mice were randomly divided into groups (*n* = 6) on day 7. 5-week-old female C3H/HeN mice were subcutaneously injected with 3 × 10^6^ SCC7 cells, and tumor-bearing mice were randomly divided into groups on day 15. Once the tumor volume reached 100 to 200 mm^3^, the mice were subjected to treatments as described in the main text. Tumor sizes were measured and calculated based on the following formula: length × width^2^ × 0.5.

### Immunohistochemistry (IHC)

For IHC, tumor tissues were isolated on day 10 post-treatment and fixed in 4% paraformaldehyde, followed by paraffin embedding. Following deparaffinization, the tissue sections were treated with 3% hydrogen peroxide to inhibit endogenous peroxidase activity, and subsequently blocked with 5% goat serum. Primary antibodies were applied and incubated overnight at 4 °C. The sections were then treated with streptavidin-HRP for 1 h and stained with a diaminobenzidine substrate. Hematoxylin was employed for counterstaining. After rehydration and mounting, the sections were observed and photographed using a microscope.

### Immunofluorescence assay (IFA)

Fresh tissues were dehydrated overnight in 30% sucrose solution until sedimentation occurred. Dehydrated tissues were blotted dry with filter paper and embedded in Optimal Cutting Temperature (OCT) compound within cryomolds. To minimize bubble formation, OCT was gently dispensed around the tissue. Molds were horizontally immersed in liquid nitrogen for rapid freezing, ensuring no liquid nitrogen infiltration. Frozen blocks were stored at −20 °C for 10 min to stabilize. A cryostat (Leica CM1950) was pre-cooled to −20 °C prior to sectioning. Serial sections (5-μm thickness) were cut using chilled forceps and blades. Sections were temporarily stored on ice or preserved at −80 °C for long-term use.

Sections were equilibrated at room temperature for 30 min, followed by three PBS washes (3 min each). Permeabilization was performed with 0.2% Triton X-100 for 10 min and washed thrice with PBS. Tissues were blocked with 5% bovine serum albumin (BSA) for 1 h. Primary antibodies (diluted in blocking buffer) were applied to sections and incubated at room temperature for 2 h. After three PBS washes, fluorescence-conjugated secondary antibodies were added and incubated at 37 °C for 1 h under light-protected conditions. Following final PBS washes, slides were air-dried and mounted with DAPI-containing antifade medium.

### Gene set enrichment analysis

Gene Ontology (GO) enrichment analysis was performed using the cluster Profiler package in R (4.4.2). Statistical significance was determined using hypergeometric tests with a *p*-value threshold of 0.05. The results were visualized with bar charts using the ggplot2 package.

### CIBERSORT analysis

Samples were carried out according to the CIBERSORT instructions (https://cibersort.stanford.edu). Patients were categorized into high and low SUV39H2 expression groups based on the median expression. Gene expression data were then input into the CIBERSORT algorithm to analyze the differences in the proportion of infiltrating immune cell types between the groups. The results were visualized using box plots. A significance threshold of *p* < 0.05 was applied to identify differences in immune cell composition between the groups.

### Statistical analysis

The results are presented from at least three independent experiments. Statistical analysis was performed using the GraphPad Prism (version 9.5). The mean ± SEM was used for the description of data variability. For all analyses, two-tailed unpaired Student’s t test for comparisons between two independent groups, while One-way ANOVA was applied for comparisons involving more than two groups. *p* < 0.05 was considered as statistically significant, ns is no statistical difference.

## Supplementary information

Supplementary Figure legends

Original Western blots

Figure S1

Figure S2

Figure S3

Figure S4

## Data Availability

All data supporting the findings of this study are available in the paper and its Supplementary Information. Data are available in the public, open access repositories from The Cancer Genome Atlas Data Portal (https://www.cancer.gov/tcga/) and the Gene Expression Omnibus (https://www.ncbi.nlm.nih.gov/geo/, GSE223085). The CRISPR screening datasets generated during the current study have not yet been deposited in a public repository but are available from the corresponding author upon reasonable request. The datasets used and analyzed during the current study, as well as all relevant data, are available from the corresponding author upon reasonable request and are included in the article or provided as supplementary information.
