# Supplementary material for: CRISPR/Cas9 screening identifies SUV39H2 as a key regulator of oHSV-1 resistance in oral squamous cell carcinoma
Source: Cell Death Discov. 2025 Aug 23;11:402. doi: 10.1038/s41420-025-02702-7 (PMC12375032; doi:10.1038/s41420-025-02702-7)
Supplement: Supplementary file 1 — Supplementary Figure legends [file 41420_2025_2702_MOESM1_ESM.docx]

**Figure S1. SUV39H2 overexpression inhibited oHSV-1 replication in SCC15 cells.**

A. Western blotting analysis demonstrated successful overexpression of SUV39H2 in SCC15 cells, with tubulin used as a loading control. B. SCC15 cells were infected with oHSV-1 at MOI of 0.03 for 30 h and 0.05 for 24 h. Fluorescence microscopy was employed to assess the replication efficiency of oHSV-1 in SCC15 cells overexpressing SUV39H2. C. Quantification of viral yields in SCC15 control cells (CON) and SUV39H2-overexpressed SCC15 cells (SUV39H2^OE^). D. Examination of viral protein levels in CON and SUV39H2^OE^ SCC15 cells. E. Effect of SUV39H2 on oHSV-1 growth. Quantification of the virus was performed at the indicated intervals using standard plaque assays on Vero cells. Data are expressed as the mean ± SEM. **P* < 0.05, ***P* < 0.01, ****P* < 0.001.

**Figure S2. Generation of T-VEC.**

A. Schematic diagram of T-VEC. (B) SCC15 cells were infected with T-VEC at an MOI of 0.1 or transfected with pCDH-GM-CSF (0.5 μg) for 48 h. Supernatants were then collected, and GM-CSF concentration was measured with ELISA. Data are expressed as the mean ± SEM. ****P* < 0.001.

**Figure S3 Effects of OTS186935 pretreatment on T-VEC replication in SCC15 cells.**

A. SCC15 cells were treated with OTS186935 (0.1 µM and 0.5 µM) for 24 h and then infected with T-VEC at an MOI of 0.03 for an additional 30 h. The cell culture supernatants were collected and quantified by titration on Vero cells. B. Expression levels of viral proteins were assessed by western blotting. Tubulin serves as a loading control. Data are expressed as the mean ± SEM. ****P* < 0.001.

**Figure S4 Dose–response proliferation curves of SCC15, SCC7, and MCF7 cell lines.**

A. SCC15, SCC7, and MCF7 cell lines were treated with OTS186935 for 24 h. Cell viability was assayed using the CCK8 kit and normalized to DMSO-treated controls.

B, D. Cell viability was measured at 24 h, 48 h, and 72 h using the CCK-8 assay in NC and shSUV39H2 cells lines (SCC15 and SCC7). Data are presented as the mean ±SEM from three independent experiments.
