# Supplementary figures and images for: CRISPR/Cas9 screening identifies SUV39H2 as a key regulator of oHSV-1 resistance in oral squamous cell carcinoma
Source: Cell Death Discov. 2025 Aug 23;11:402. doi: 10.1038/s41420-025-02702-7 (PMC12375032; doi:10.1038/s41420-025-02702-7)

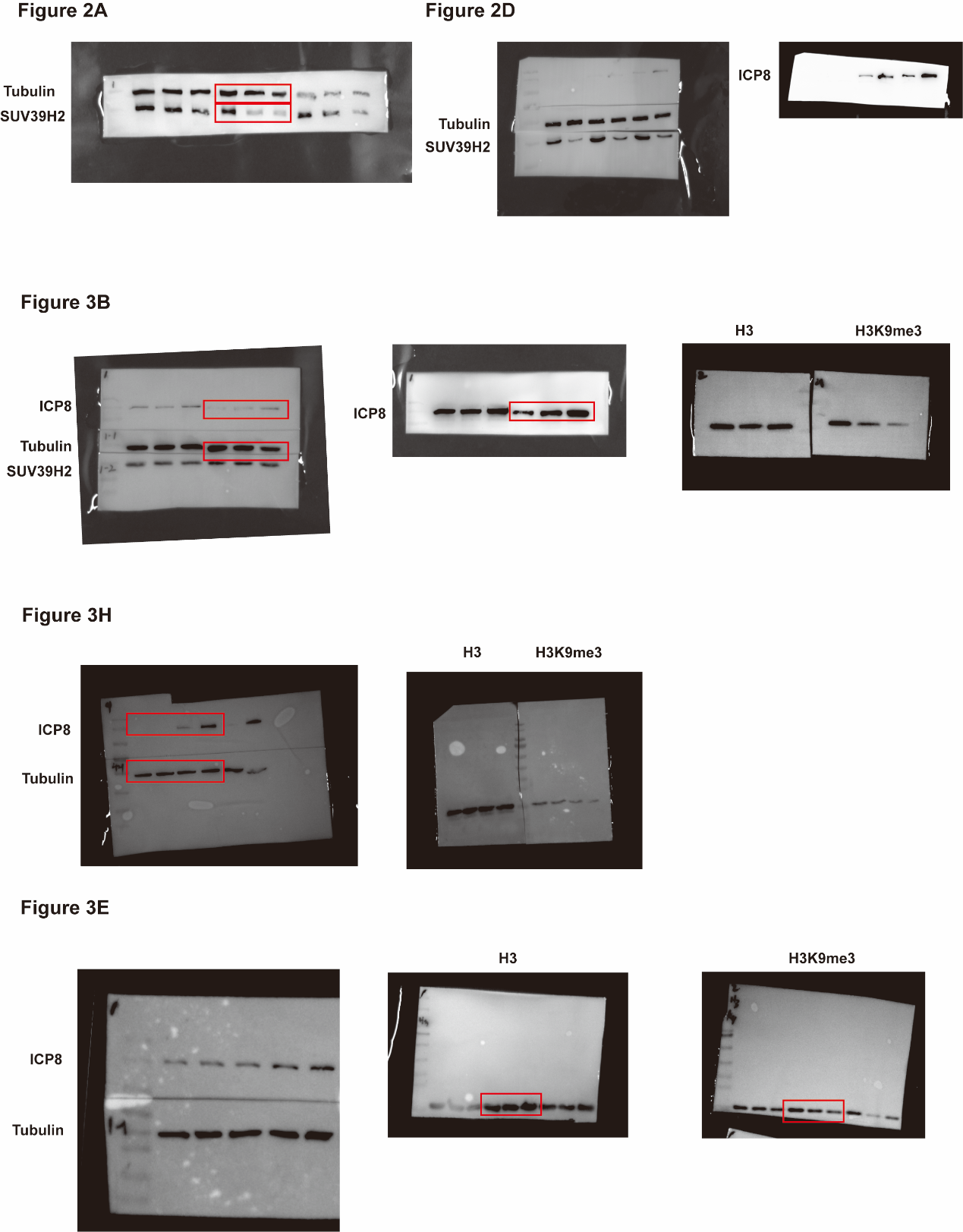


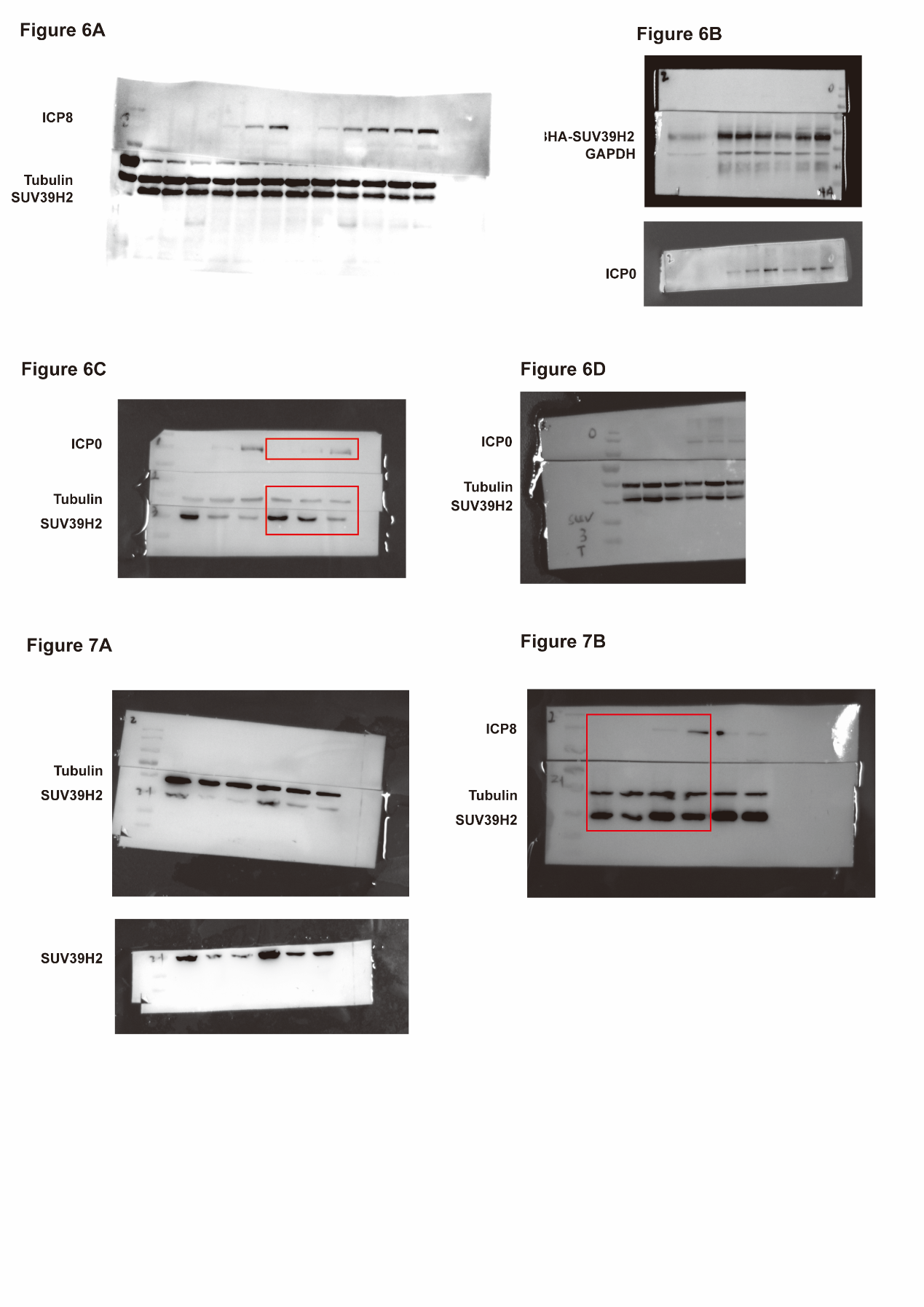


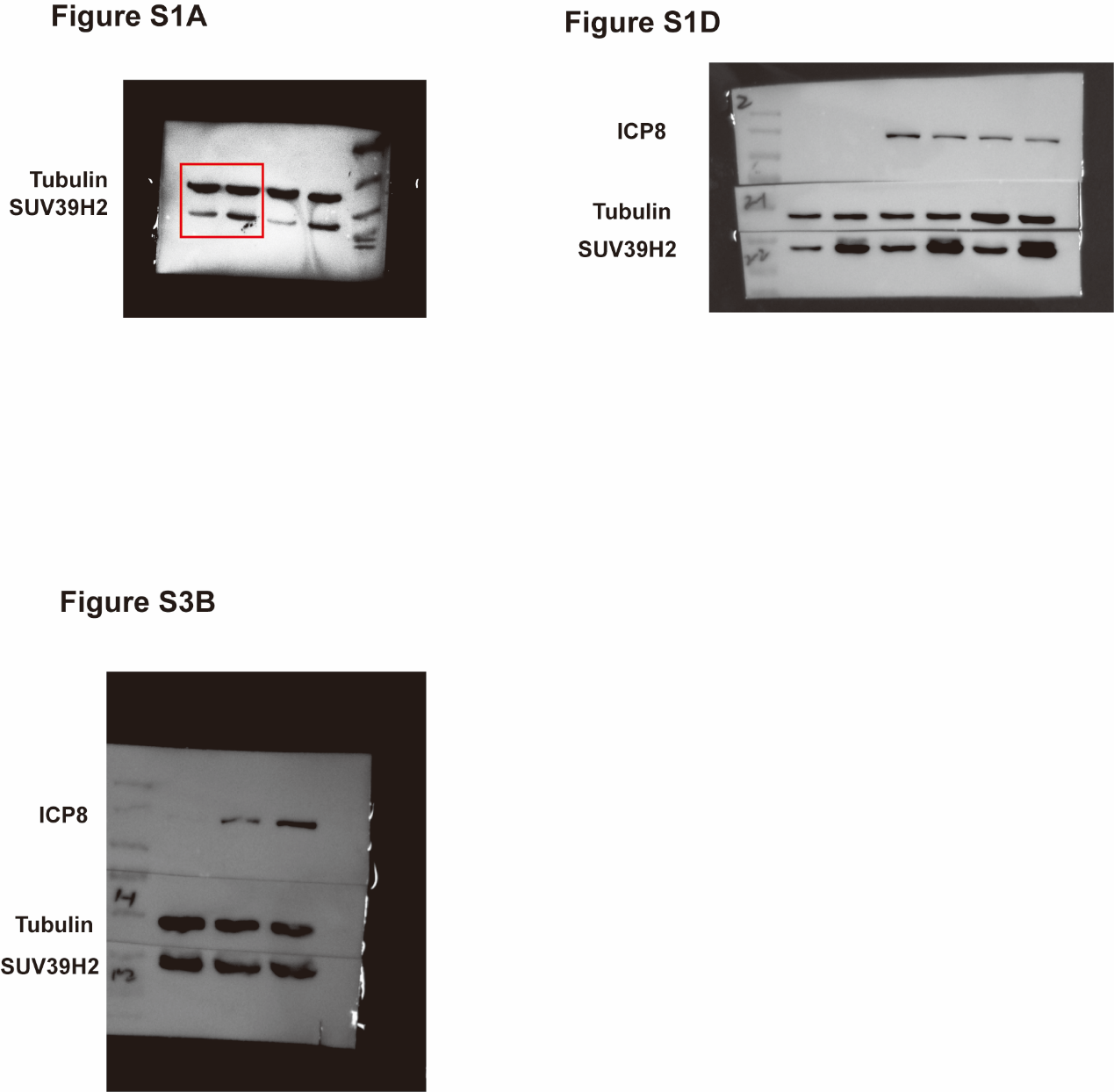

Supplement: Supplementary file 2 — Original Western blots [file 41420_2025_2702_MOESM2_ESM.docx]

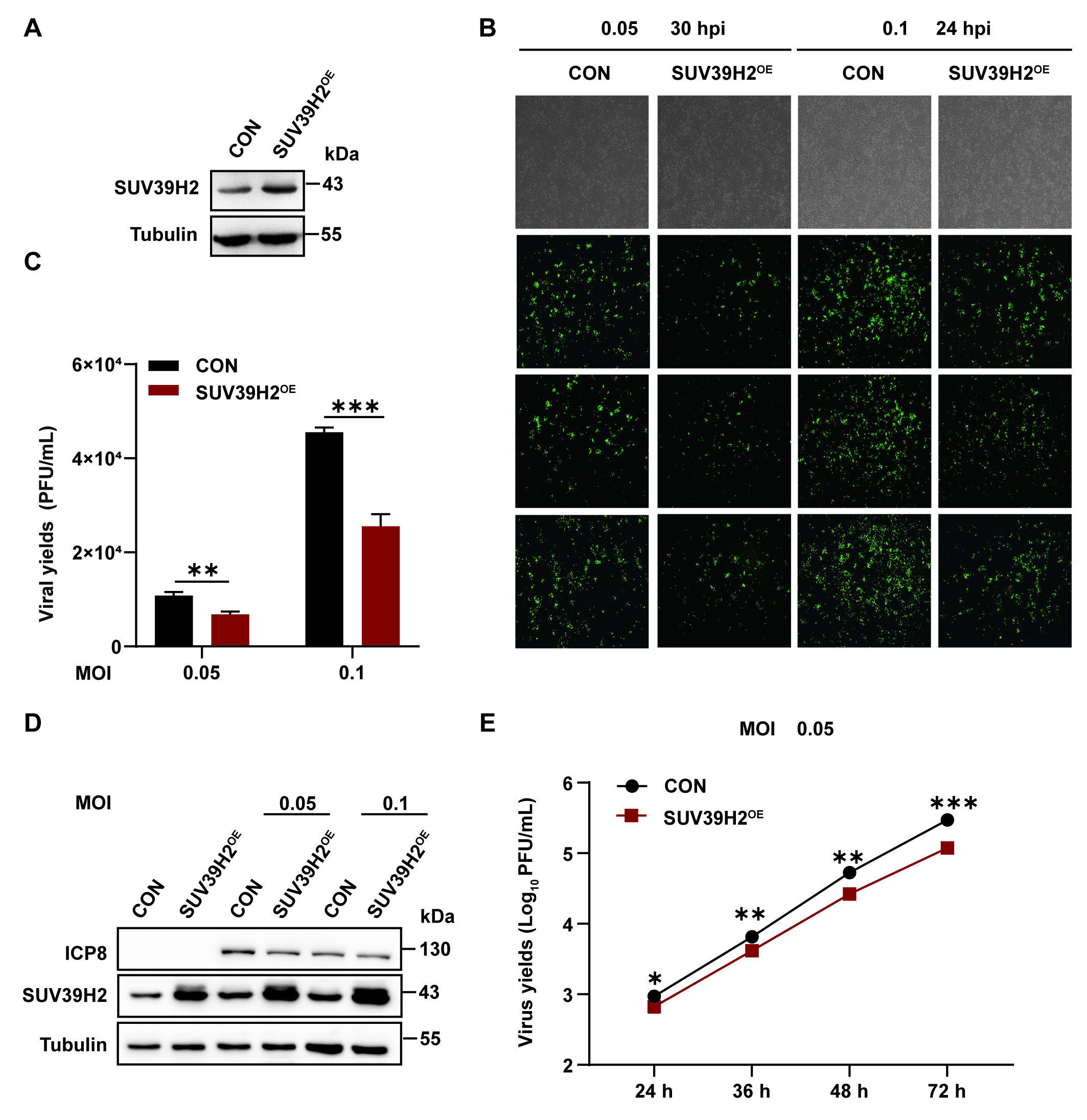

Supplement: Supplementary file 3 — Figure S1 [file 41420_2025_2702_MOESM3_ESM.tif]

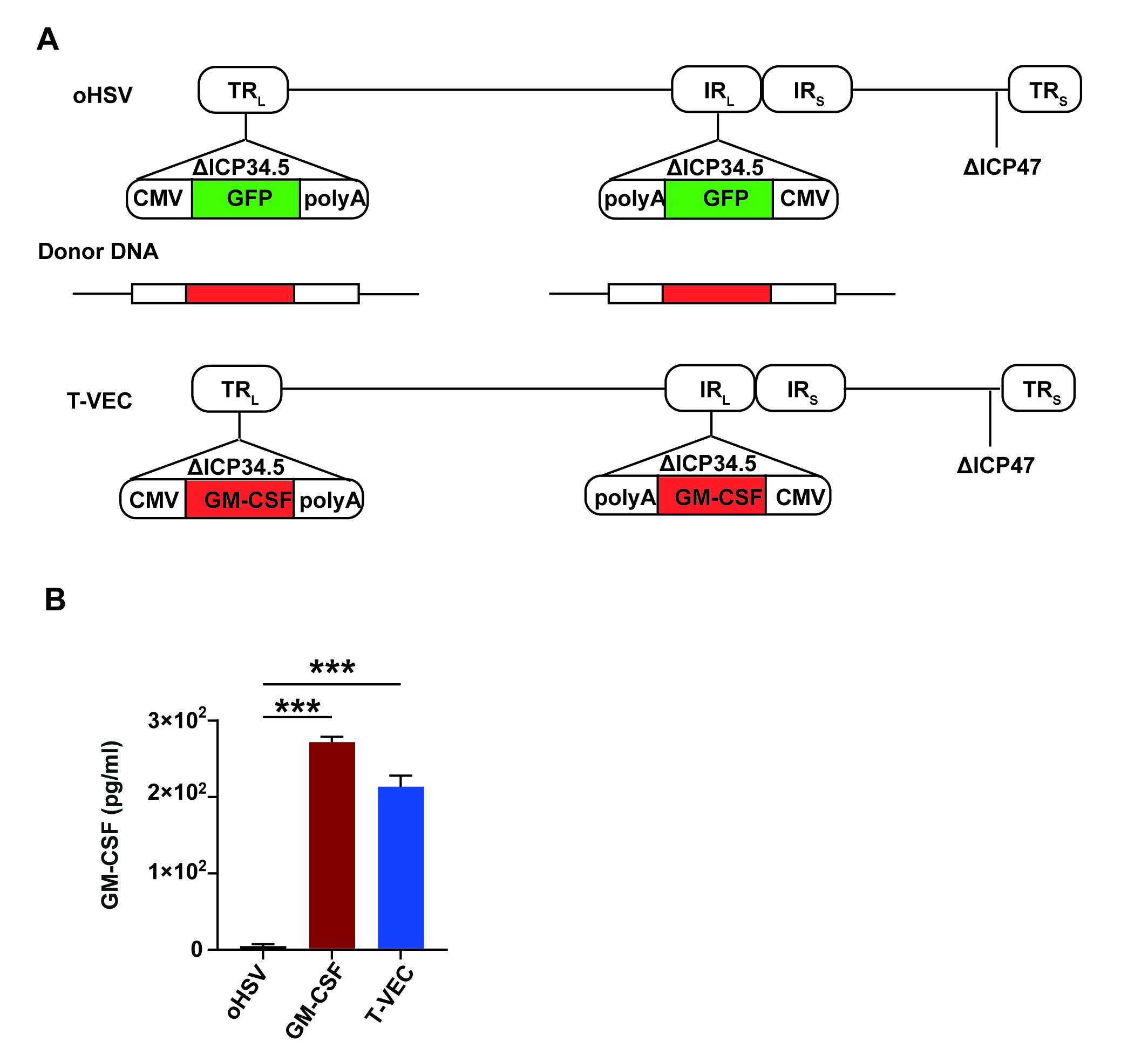

Supplement: Supplementary file 4 — Figure S2 [file 41420_2025_2702_MOESM4_ESM.tif]

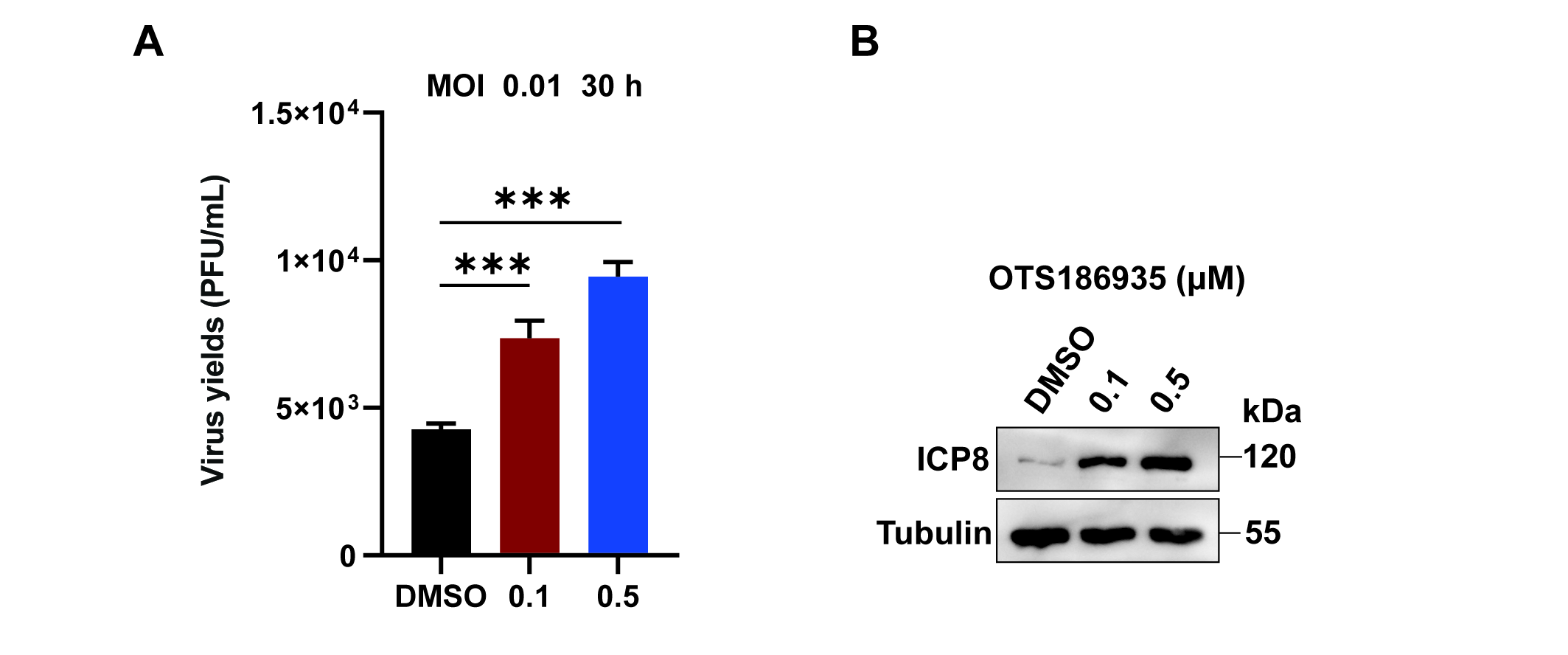

Supplement: Supplementary file 5 — Figure S3 [file 41420_2025_2702_MOESM5_ESM.tif]

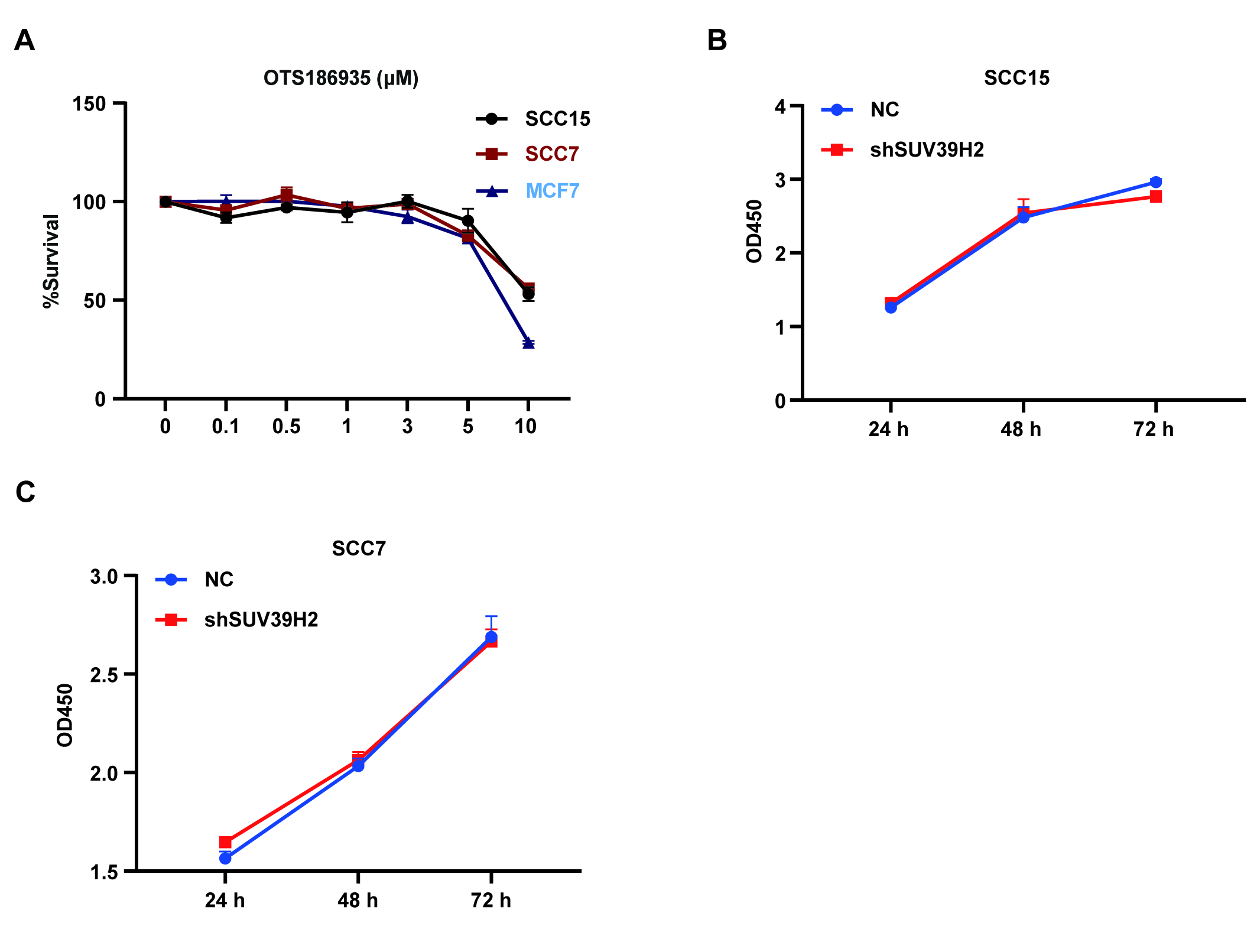

Supplement: Supplementary file 6 — Figure S4 [file 41420_2025_2702_MOESM6_ESM.tif]
